# Current Knowledge about the New Drug Firibastat in Arterial Hypertension

**DOI:** 10.3390/ijms23031459

**Published:** 2022-01-27

**Authors:** Emma Hansen, Daniela Grimm, Markus Wehland

**Affiliations:** 1 Department of Biomedicine, Aarhus University, Ole Worms Allé 4, 8000 Aarhus, Denmark; 201904581@post.au.dk (E.H.); dgg@biomed.au.dk (D.G.); 2 Department of Microgravity and Translational Regenerative Medicine, Otto von Guericke University, Universitätsplatz 2, Building 28, 39106 Magdeburg, Germany

**Keywords:** firibastat, hypertension, brain RAS

## Abstract

Hypertension significantly increases the risk of cardiovascular disease. Currently, effective standard pharmacological treatment is available in the form of diuretics, ACE inhibitors, angiotensin II receptor blockers and calcium channel blockers. These all help to decrease blood pressure in hypertensive patients, each with their own mechanism. Recently, firibastat, a new first-in-class antihypertensive drug has been developed. Firibastat is a prodrug that when crossing the blood-brain barrier, is cleaved into two active EC33 molecules. EC33 is the active molecule that inhibits the enzyme aminopeptidase A. Aminopeptidase A converts angiotensin II to angiotensin III. Angiotensin III usually has three central mechanisms that increase blood pressure, so by inhibiting this enzyme activity, a decrease in blood pressure is seen. Firibastat is an antihypertensive drug that affects the brain renin angiotensin system by inhibiting aminopeptidase A. Clinical trials with firibastat have been performed in animals and humans. No severe adverse effects related to firibastat treatment have been reported. Results from studies show that firibastat is generally well tolerated and safe to use in hypertensive patients. The aim of this review is to investigate the current knowledge about firibastat in the treatment of hypertension.

## 1. Introduction

Arterial hypertension is one of the major risk factors in the development of cardiovascular disease. It is estimated by the World Health Organization (WHO) that 47% of ischemic heart disease cases and 54% of strokes are the consequence of hypertension (HT) [1]. Most often HT does not cause any symptoms, but the risk of serious cardiovascular complications such as myocardial infarction or stroke increases if a patient has high, uncontrolled blood pressure [2]. Patients diagnosed with HT are treated in different ways. By lowering blood pressure (BP), the risks associated with HT can be reduced. BP can be lowered by nonpharmacological options and by pharmacological intervention [3]. Quantum Genomics is a biopharmaceutical company that over the past few years, has developed the new first-in-class drug firibastat as a treatment for HT. Firibastat is an antihypertensive drug that targets the brain renin–angiotensin system (RAS) [4]. This review will investigate the current knowledge about firibastat in HT, its mechanisms of action and pharmacological effects.

## 2. Arterial Hypertension

Arterial hypertension is defined by the WHO as a condition where the pressure in the blood vessels (arteries) is persistently raised [5]. BP is determined by cardiac output and peripheral vascular resistance. High BP occurs if one or a combination of both factors are elevated [1]. BP is given in mmHg (systolic and diastolic). According to the European Society of Hypertension, the exact definition of arterial HT is defined as systolic blood pressure (SBP) ≥ 140 mmHg and diastolic blood pressure (DBP) ≥ 90 mmHg [6,7]. HT is divided into different categories depending on how elevated the BP is. Values below 120 mmHg for systolic BP and below 80 mmHg for diastolic BP are considered optimal. Normal BP ranges from 120–129/80–84 mmHg, high normal from 130–139/85–69 mmHg. Hypertension begins with grade 1 from 140–159/90–99 mmHg, grade 2 ranges from 160–170/100–109 mmHg, and values above 179/110 mmHg constitute grade 3 hypertension. Patients with over 140 mmHg systolic BP and below 90 mmHg diastolic BP suffer from isolated systolic blood pressure [6,7,8]. 

### 2.1. Causes

Primary hypertension and secondary hypertension (SH) are the two main categories of HT. Primary hypertension is also known as essential hypertension (EH) and accounts for 95% of all cases [8]. The causes of EH are still not identifiable but tend to develop over years [2]. EH is a complex multifactorial disease with genetic and environmental components. Risk factors such as obesity, chronic inflammation, genetic predisposition, gender, age, increased sodium intake and lifestyle behavior in general all contribute to the development of HT [9]. SH is caused by an underlying condition. Among adults, SH most often develops as a consequence of another disease. The most common causes of SH are renal failure, obstructive sleep apnea, Cushing syndrome, hyperaldosteronism, and atherosclerotic renal artery stenosis [2,10].

BP is controlled by different mechanisms. The sympathetic nervous system (SNS), renin–angiotensin–aldosterone system (RAAS) and neuropeptides all play an important role in controlling BP. An imbalance/disturbance in these control mechanisms will lead to an increased vascular volume and a decreased excretion of sodium in the kidney. A shift in the pressure–natriuresis relationship is a pivotal component in the pathogenesis of HT [9].

### 2.2. Mortality and Prevalence

HT is one of the leading causes of premature death worldwide and many countries are struggling to fight HT. It is estimated by the WHO that 1.28 billion people worldwide (aged 30–79) have HT. This number has increased from 594 million in 1975. The majority of these people live in low- and middle-income countries, and 46% of people with HT are unaware of the problem because HT does not usually cause any symptoms [11]. The prevalence of HT depends on the country, for example, in the United States the prevalence of HT in 2017–2018 among adults aged ≥18 was 45.4%. Adjusted for sex, the prevalence was 39.7% among women and 51% among men. In addition, it has been shown that factors such as ethnic origin and level of education also have an influence on prevalence [12]. It is estimated that by 2025, 1.5 billion people worldwide will suffer from HT [6,13].

Arterial hypertension is a cause of premature death and is associated with an increased risk of cardiovascular disease (CVD) [14]. HT-related CVD deaths in the United States between 2000 and 2018 increased from 171,259 to 270,839 (+0.5%/year) [15]. Every year, 7.6 million people worldwide die with HT as the responsible risk factor. It is estimated that 60 million people worldwide die each year, meaning that approximately 13.5% of all deaths are HT-related [16].

### 2.3. Diagnosis

The diagnosis of HT is most often based on repeated BP measurements with a BP monitor in a clinical office. It is mostly based on these clinical measurements since HT is often asymptomatic. Structured population screening programs helps to detect people who are unaware that they have HT [6]. The measurements are done by placing the BP monitor with an arm cuff on the upper arm at heart level. The monitor measures both the systolic and the diastolic pressure as well as the heart rate. It is important that the patient is placed in a relaxed position and has been sitting for at least 5 min. The measurement is repeated 2–3 times with a time interval of 1–2 min, preferably on both arms [17]. Taking BP measurements in lying and standing positions should be considered, especially when it comes to older people, people with diabetes or another condition in which orthostatic hypertension may occur [6]. To get the most accurate measurement, the average of the three readings is determined. This average makes it possible to categorize the stage of arterial hypertension. If the measurements indicate HT, the doctor will recommend other tests to confirm the diagnosis and check for any underlying conditions. Ambulatory/home BP monitoring (ABPM/HBPM), blood tests, urine tests and EKGs help to confirm the diagnosis [2]. In addition to the measurements, the diagnosis should also include an assessment of CDV risk, features associated with SH, target organ damage and gene mutations [18,19]. White-coat HT is a term used when BP is elevated in the clinical office, but not outside of the office. Masked HT is elevated BP at home and normal clinical office readings. ABPM and HBPM are out-of-office measurements and provide a larger number of measurements, which are considered to be more representative of daily life [6]. In an observational study, the aim was to determine the number and timing of measurements needed to give a trustworthy and reliable mean BP to give the most accurate HT diagnosis. Results from this study showed that measurements taken in the afternoon were the most accurate compared to the 24 h BP mean. In addition, it was found that 8–10 BP readings are enough to give the diagnosis [20].

## 3. Standard Treatment Options for Arterial Hypertension

When HT is diagnosed, usually the first recommendation for the patient is to make some lifestyle changes including eating a healthy diet, reducing the sodium intake, limiting alcohol consumption and increasing the amount of physical activity. Sometimes these changes are not enough to reduce the BP. In these cases, several pharmacological treatment options are used [2]. Pharmacological treatment options include drugs such as diuretics, ACE-inhibitors, angiotensin-II-receptor blockers, and calcium channel antagonists. When monotherapy is not sufficient to reduce the BP, two or three of the antihypertensive medications are combined in a double or triple combination therapy [6,18]. In the following subchapters, a short overview of the standard pharmacological treatment options of HT is given.

### 3.1. Diuretics

Diuretics have an antihypertensive effect because they reduce the blood volume. There are three main groups of diuretics: loop diuretics, thiazides and potassium-sparing diuretics. In some cases, different types of diuretics are combined in treatment [21]. Loop diuretics work by inhibiting the sodium-potassium-chloride symporter in the loop of Henle in the kidneys. Loop diuretics are often used to treat edema. Examples of loop diuretics are furosemide, ethacrynic acid and torsemide [22]. Thiazides promote natriuresis and diuresis by blocking the sodium-chloride (Na/Cl) co-transporter in the proximal segment of the distal convoluted tubuli. Thiazides help to eliminate water and sodium from the body, thereby lowering the blood volume, which leads to a reduced BP. A commonly used thiazide is hydrochlorothiazide [23]. Amiloride is an example of a potassium-sparing diuretic. It acts on the sodium channels of the renal epithelial cells. Potassium-sparing diuretics are often used in combination with loop diuretics or thiazides to treat edema and HT, primarily to limit potassium loss [24].

### 3.2. Angiotensin Converting Enzyme (ACE) Inhibitors

ACE-inhibitors act on the RAAS. They inhibit the production of angiotensin II (Ang-II). By inhibiting the production of Ang-II, the downstream effects of Ang-II are inhibited as well. When Ang-II is inhibited, the systemic vascular resistance is reduced. Ang-II, when not inhibited, has a strong constricting effect on the small arteries. Captopril is an ACE-inhibitor and became available in 1981. Other examples are enalapril and ramipril [18,25].

### 3.3. Angiotensin-II-Receptor Blockers

Angiotensin-II-receptor blockers (ARB) also act on the RAAS and have similar effects to the ACE-inhibitors. ARBs do not inhibit the production of Ang-II but block its action. This is achieved by its binding to the Ang-II type 1 receptor. Losartan is the first ARB and was launched in 1995. Other ARBs are candesartan and irbesartan [25,26]. Because they have similar effects to the ACE-inhibitors, the two treatment options should not be combined [6,26].

### 3.4. Calcium Channel Blockers

Calcium channel blockers block the voltage-gated L-type calcium channels, which results in the relaxation of the vascular smooth muscle cells. This relaxation causes vasodilation and thereby reduces the BP. In cardiac muscle cells, the contractility is reduced [27]. Calcium channel blockers can easily be combined with other antihypertensive treatment options. A common side effect of calcium channel blockers is peripheral edema, especially in obese patients [18]. Amlodipine is a calcium channel blocker used to treat HT. Other examples of calcium blockers are nifedipine, felodipine and nisoldipine [27].

### 3.5. Beta-Adrenoceptor Antagonists (BAAs)

BAAs have recently been relegated from a first to a second line therapy, yet in combination they are still widely used and effective. The β adrenoceptors (AR) β_1_, β_2_, and β_3_ mediate heart rate, contractility and conduction velocity in cardiac tissue. In peripheral blood vessels, β_2_-AR is predominant, influencing vasodilation. β_3_-AR is mainly found in the gastrointestinal tract and in adipose tissue, where it regulates intestinal muscle tension and lipolysis. BAAs can be classified into three generations. Generation 1 comprises the non-selective BAAs such as propranolol. The cardioselective (β_1_-AR-selective) BAAs constitute generation 2 with such drugs as bisoprolol or metoprolol. Third-generation BAAs have various cardioselectivities, but possess additional cardiovascular-related properties, such as a vasodilative activity. Nebivolol, carvedilol or labetalol are members of this group [28,29,30].

## 4. Firibastat

Firibastat is a new, centrally-acting drug developed by Quantum Genomics to treat high BP. It is used in the prevention of CVD risks. The main purpose of firibastat is to target the brain RAS [31], in contrast to other antihypertensive treatment options that target the systemic RAAS. It has been found that overactivity in the brain RAS can be related to the development and maintenance of HT. Therefore, the brain RAS is a possible therapeutic treatment target when it comes to HT [32]. All members of the systemic RAAS are present in the brain RAS, which include the precursor angiotensinogen, enzymes, peptides, and angiotensin receptors [33].

### 4.1. Mechanism of Action

**Figure 1:**
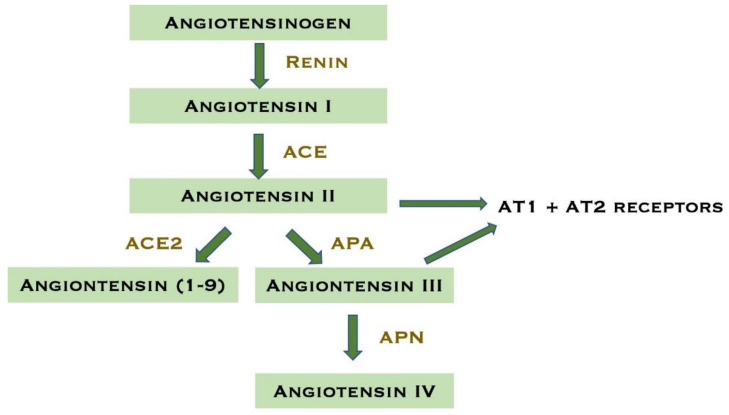
Schematic diagram of the brain renin–angiotensin system. Abbreviations: Angiotensin-converting enzyme (ACE), angiotensin-converting enzyme type 2 (ACE2), aminopeptidase A (APA), aminopeptidase N (APN), angiotensin receptor type 1 (AT1), angiotensin receptor type 2 (AT2). Modified for this review from [35].

Firibastat is an aminopeptidase A (APA) inhibitor. APA plays an important role in the brain RAS where it hydrolyses the N-terminal aspartate residue from Ang-II to angiotensin-III (Ang-III) [34]. APA is a membrane-bound zinc metalloprotease. Another membrane-bound zinc metalloprotease is aminopeptidase N (APN). APN cleaves the N-terminal of Ang-III to generate angiotensin IV (Ang-IV) [32]. Figure 1 shows a simplified schematic overview of the brain RAS. 

**Figure 2:**
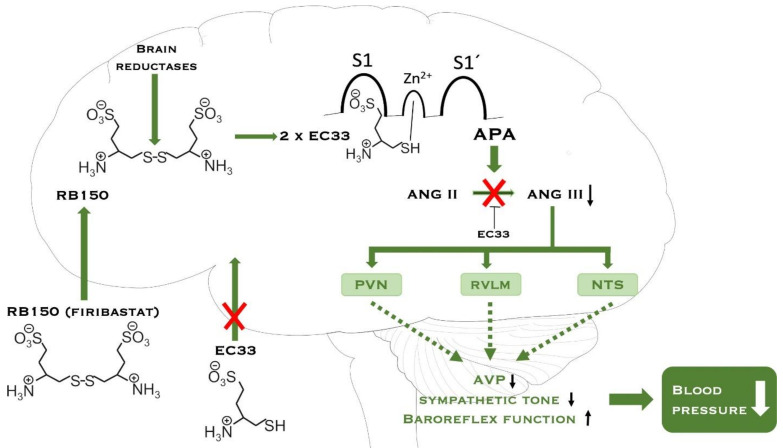
The prodrug RB150 (firibastat) can cross the blood–brain barrier after oral administration because of the disulfide bridge. RB150 consists of 2 EC33 molecules. EC33 cannot cross the blood–brain barrier. When RB150 has entered the brain, two active molecules of EC33 are released by brain reductases. The active EC33 molecules binds to the active site of aminopeptidase A (APA) and inhibits the formation of angiotensin III (Ang-III). Ang-III is the effector peptide. Normally Ang-III increases the blood pressure via 3 different mechanisms: (1) increases the release of arginine-vasopressin (AVP), (2) sympathetic nerve activation, and (3) inhibition of the baroreflex. When APA is inhibited, angiotensin II is not cleaved into Ang-III, which exerts a stimulatory action on the control of blood pressure in brain structures, resulting in decreased blood pressure. Abbreviations: aminopeptidase A (APA), angiotensin II (Ang-II), angiotensin III (Ang-III), paraventricular nucleus (PVN), rostral ventrolateral medulla (RVLM), nucleus of the solitary tract (NTS), arginine-vasopressin (AVP). Modified from [35].

Ang-II and Ang-III exhibit a similar affinity for angiotensin type 1 receptor (AT1) and angiotensin type 2 receptor (AT2). Binding to these receptors increases BP through three mechanisms: increased release of arginine-vasopressin (AVP), sympathetic nerve activation and inhibition of the baroreflex. In a study by Mark et al., it was found that by inhibiting APA activity and the formation of Ang-III with firibastat, a dose-dependent decrease in BP occurred in rats [32,36]. EC33 ((3S)-3-amino-4-sulfanyl-butane-1-sulfonic acid) is an APA inhibitor. When EC33 binds to APA it inhibits the conversion of Ang-II to Ang-III. EC33 is unable to cross the gastrointestinal, liver and blood–brain barrier (BBB), when taken orally. For this reason, a prodrug of EC33 has been manufactured. This prodrug consists of two EC33 molecules connected by a disulfide bridge. The prodrug is called RB150 (4,4-dithio bis(3S)-3-aminobutyl sulfonic acid), which was later named firibastat. The thiol group in RB150 is engaged in the disulfide bridge and is unable to interact with the zinc atom in active site of APA. By binding two EC33 molecules together to form RB150, it is possible to cross the gastrointestinal, liver and BBB. Once the RB150 has crossed the BBB, it is cleaved by brain reductases into two active EC33 molecules. These two EC33 molecules are now able to bind to APA’s active site and inhibit the conversion of Ang-II to Ang-III, and thus Ang-III systemic effects [32,36]. Figure 2 shows how firibastat affects the brain RAS and thereby decreases the BP.

Although Ang-II and Ang-III have similar effects, studies in murine brains have attempted to define their respective roles in relation to cardiovascular function. An intra-cerebroventricular injection of EC33 in hypertensive rats showed a decrease in blood pressure due to the inhibition of APA. An intravenous injection, however, showed no decrease, indicating that EC33-induced BP decrease is a central but not systemic effect. These findings suggest that Ang-III is an important effector peptide of the brain RAS and in the control of BP [37,38]. The conversion from Ang-II to Ang-III is central to increase BP, therefore it is a potential therapeutic target whereby this conversion is inhibited. A possible explanation for the fact that Ang-II does not induce a BP increase under brain APA blockade might be that in the presence of an APA inhibitor, alternative pathways of Ang-II are activated via other peptidases such as ACE2, aminopeptidases or endopeptidases, leading to Ang3–8, Ang1–5, Ang4–8, and Ang1–7, all of which do not activate the AT1 receptor [39,40,41]. This hypothesis is further supported by observations by Feng et al., and Yamazato et al., who showed that ACE2 overexpression in the brain can prevent low-dose Ang-II-induced hypertension, and reduces BP in SHR rats, respectively [42,43].

### 4.2. Indications

Firibastat is a drug that can be used in the treatment of HT. The inhibitory effect on APA has been shown to decrease BP, which is favorable for hypertensive patients. The presence of high BP is therefore an indication for using firibastat as a pharmacological treatment option. It was found that firibastat has a particularly great effect in black and obese people dealing with resistant HT. Studies have shown that BP significantly decreases in these groups. Firibastat is an alternative or complementary option to treat HT when other standard monotherapy treatment methods are no longer effective [32,44]. In addition, firibastat can also be used in the prevention or risk-taking treatment of cardiac dysfunction after myocardial infarction (tested in mice) [45]. Finally, it has been suggested that firibastat may become an important drug to treat resistant HT by affecting brain RAS [46].

### 4.3. Pharmacokinetics

The pharmacokinetics of firibastat have been studied in clinical trials. Table 1 provides an overview of plasma pharmacokinetic parameters for firibastat and EC33 when different doses (125, 500 and 1250 mg) were applied [4].

### 4.4. Interactions

Firibastat is a new drug and is therefore still being tested in various clinical trials. Therefore, there is currently limited knowledge about the interactions between firibastat and other drugs. However, there is a study that was performed before phase 1 where the effect of firibastat was tested in rats and dogs. This study showed that none of the human recombinant cytochrome P450 enzymes were significantly inhibited by either RB150 or EC33. The human recombinants involve CYP1A2, CYP2C9, CYP2C19, CYP2D6 and CYP3A4 and were all tested in vitro at 10 μmol/L in fluorimetric substrate assays. Based on this experiment, strong inducers of these specific human recombinant cytochrome P450 enzymes will not interact with firibastat [4].

### 4.5. Animal Studies

The first studies in rats showed that a direct injection of EC33 into the cerebral ventricles of normotensive Wistar-Kyoto (WKY) rats and spontaneously hypertensive rats (SHR) leads to a specific inhibition of APA activity and a dose-dependent decrease in BP. It was also shown that i.v. injection of EC33 did not have these effects [38]. Oral administration of the prodrug RB150, which is able to cross the blood–brain barrier, in conscious spontaneously hypertensive rats inhibited brain APA activity and dose-dependently reduced BP without influencing systemic RAS activity [36]. Similar effects were observed when hypertensive deoxycorticosterone acetate (DOCA)-salt rats were given RB150 (50 mg/kg per day) over 24 days. The animals showed a significant drop in SBP and did not develop tolerance to RB150 over the treatment period [32]. Furthermore, RB150 in conscious DOCA-salt rats reduced brain APA activity to levels comparable to those in normotensive rats. The induced drop in BP occurred in under 2 h and lasted for several hours [47].

## 5. Clinical Trials with Firibastat

In 2012, RB150 was tested for toxicity, safety and pharmacokinetics in rats and dogs. The study demonstrated that RB150 is well tolerated in both dogs and rats. Doses of RB150 up to 1000 mg/kg were tested for 28 days and showed no signs of increased mortality. With these results, it has been decided to investigate the possibilities of firibastat in relation to HT with human trials [36]. Table 2 provides an overview of the clinical trials with RB150/firibastat/QGC001 in relation to HT in humans.

### 5.1. Safety and Adverse Effects of Firibastat

In general, firibastat has been very well tolerated in both the Phase 1 and Phase 2 clinical trials. During Phase 1 (NCT01900171), vital signs (for example, BP, heart rate, oral temperature and ECG) were measured at regular intervals from day 1 to day 11 to ensure safety and tolerance among the subjects. In Phase 1, no serious adverse effects (AEs) were reported. Only six mild to moderate AEs were reported in 5 of 42 subjects (11.9%) in the firibastat group. Only one of the five reported AEs was associated with firibastat treatment. The person experienced mild asymptomatic orthostatic hypertension 6 h after ingestion of 500 mg firibastat. No clinically significant abnormalities were found during the Phase 1 study [4].

In NCT02322450 (Phase IIa), there were 3 out of 34 subjects who experienced serious AEs in the group treated with firibastat. The AEs were vestibular disorder (lasted less than 24 h), skin rash, and moderate arthralgia. No changes in the biochemical safety parameters were detected [48].

In NEW-HOPE (Phase IIb) (NCT03198793), a total of 107 subjects experienced AEs, but only 36 (14.1%) of them were related to firibastat treatment. A total of 19 people (7.5%) stopped treatment due to AEs. The most common AEs were headache (4.3%) and skin reactions (3.1%). Five serious adverse events were reported but only one was related to firibastat treatment, a case of erythema multiforme. The general tendency of firibastat to induce skin lesions of various severity as AEs might be due to the presence of a sulfhydryl group in the EC33 molecule, as sulfhydryl drug-induced eruptions have already been described earlier [53,54]. No significant changes were measured in clinical laboratory tests from baseline to trial completion [44]. Taken together, these studies performed in humans prove that firibastat is well tolerated and safe to use in patients.

### 5.2. Firibastat vs. Standard Pharmacological Treatment

As firibastat is still a relatively new drug, it is important to clarify that a limited number of studies have been performed regarding AEs. Although many variants of antihypertensive medication exist, 45.5% of the treated patients in the USA still do not achieve the BP control goal of <140/90 mmHg. This shows that there is room for great improvement in terms of efficiency in this area [55]. Studies have shown that a combination therapy with pre-existing hypertensive drugs significantly reduces BP [55,56,57].

One of the most frequently used antihypertensive drugs in the United States and Western Europe is the diuretic hydrochlorothiazide. Recent studies show a duration and dose–response relationship between the use of hydrochlorothiazide and cutaneous squamous cell carcinoma [58]. Another study similarly showed that there was a relationship between the use of hydrochlorothiazide in high consumption and basal cell carcinoma. Hydrochlorothiazide for the treatment of HT should be considered more carefully than before after these findings [59]. A Danish study found a significant decrease in the use of hydrochlorothiazide from 2016 to 2020 after this risk was revealed in new studies [60]. Firibastat has the potential to be groundbreaking in the antihypertensive market based on the current knowledge about this drug. The clinical trials showed that there were no severe adverse effects, and that firibastat is effective in lowering BP [4,44,48,49,50]. In addition, it was found that firibastat has a successful BP-lowering effect in blacks, who generally struggle with resistant hyper- tension to standard treatment [44,61].

A clinical trial in 2021 [62] investigated the effects of firibastat, enalapril and hydrochlorothiazide on BP as a combination therapy and monotherapy in conscious hypertensive deoxycorticosterone acetate (DOCA)-salt rats. The study showed that oral administration of firibastat 30 mg/kg significantly reduced BP (−35.4 ± 5.2 mmHg), whereas administration of enalapril (10 mg/kg) or hydrochlorothiazide (10 mg/kg) alone did not show a significant decrease in BP. In the experiment, combination therapy with enalapril + hydrochlorothiazide was compared to tri-therapy with firibastat + enalapril + hydrochlorothiazide. The BP decrease was significantly greater with tri-therapy including firibastat (−63.3 ± 9.1 mmHg) and did not affect heart rate. The decrease in BP was greatest 5 h after ingestion of the tri-therapy that included firibastat + enalapril + hydrochlorothiazide, was persistent for 9 h, and disappeared after 24 h. This study shows that this combination therapy could be a new treatment option for patients with difficult-to-treat HT. Several BP-lowering mechanisms are affected by this tri-therapy. Firibastat blocks the formation of Ang-III and thereby normalizes brain RAS hyperactivity, enalapril is an ACE inhibitor, and hydrochlorothiazide is a diuretic that increases diuresis and thus lowers blood volume. This trial performed on DOCA-salt rats shows that a combination where firibastat is included in tri-therapy together with standard pharmacological treatment options, can also be used to effectively lower BP in hypertensive patients with resistant or difficult-to-treat HP [62]. Phase 3 (FRESH), which has not yet been completed, aims to investigate the use of firibastat as a treatment for resistant hypertension [51]. 

In September 2019, Quantum Genomics received positive feedback from the FDA on their Phase 3 design. The FDA requires that two studies be conducted that focus on efficacy and safety, respectively, prior to approval of the drug [63]. It will be interesting to follow the upcoming clinical trials that are planned for firibastat, both in terms of the effectiveness of the drug, but also whether so far unknown AEs are observed in new studies.

## 6. Materials

The literature used to write this review was found via online databases, clinical trials, and a few online webpages. The online databases where the literature was found include PubMed (https://pubmed.ncbi.nlm.nih.gov, last accessed 30 December 2021), Scopus (https://www.scopus.com, last accessed 30 December 2021) and clinicaltrials.com (https://clinicaltrials.gov). The literature search was conducted by using several different databases to ensure that the most relevant material was found. Original articles were found from systematic literature searches and from written reviews. The clinical trials included in this review are trials regarding the efficacy and safety of firibastat. The search process was conducted using the keywords listed in Table 3 below.

## 7. Conclusions

Firibastat is an APA inhibitor that inhibits the conversion of Ang-II to Ang-III, and thus the systemic effects of Ang-III. It is a new drug that lowers BP by affecting brain RAS. Clinical trials have shown that firibastat is an effective drug in lowering BP in both animals and humans. In addition, it is found to be generally well tolerated among subjects. Very few and harmless AEs were observed during the clinical trials. Combination therapy is effective in the treatment of HT. Firibastat is effec tive in combination therapy with an ACE inhibitor and a diuretic. A significant decrease in BP using combination therapy was observed. As a first-in-class drug, firibastat has the potential to play an important role in the treatment of resistant and difficult-to-treat HT based on the knowledge available about the drug so far.

## Figures and Tables

**Table 1:** Overview of plasma pharmacokinetic parameters for firibastat and EC33.

Parameter	Firibastat 125 mg	Firibastat 500 mg	Firibastat 1250 mg
C_max_ (ng/mL)	4.2	25.9	62.6
t_max_ (h)	0.7	1.7	3
t_1/2_ (h)	NC	1.1	1.7
CL_R_ (L/h)	135.3	92.3	66.7
Ae (%)	0.6	1.2	1.6
**Parameter**	**EC33** **125 mg**	**EC33** **500 mg**	**EC33** **1250 mg**
C_max_ (ng/mL)	15.4	56.3	77.3
t_max_ (h)	2.5	3	4
t_1/2_ (h)	NC	NC	2.8
Ae (%)	0.9	1	1.1

All values for each parameter are expressed as median. C_max_ is the peak plasma concentration, t_max_ is the time required to achieve C_max_, t_1/2_ is the elimination half-life, CL_R_ is the renal clearance of the substance, Ae is the amount of unchanged drug excreted in the urine (cumulative) and NC: not calculable. Table modified from [4].

**Table 2:** Overview of the recent clinical trials using RB150/firibastat.

Title	Design	Results	Conclusions
Phase I Study in Healthy Male Subjects Comparing QGC001 to Placebo NCT01900171 [4]	Randomized Double-blinded Placebo-controlled 56 participants Phase 1	Single oral doses from 10 to 1250 mg were all well tolerated both clinically and biologically in the 56 healthy male volunteers. RB150 and EC33 both had a peak plasma concentration (C_max_) linear with the dose. The plasma elimination half-life of RB150 was 1.6 h at all doses. No significant changes in SBP, DBP or heart rate were observed in any of the treatment groups AEs: 5 treatment-emergent AEs, only one (mild asymptomatic orthostatic hypotension) was considered related to study treatment	RB150 is well tolerated in healthy volunteers with a single oral administration up to 1250 mg. RB150 does not affect the systemic RAS. In normotensive individuals, single dose of RB150 did not affect SBP, DBP, or heart rate. No severe or life-threatening adverse effects were observed
Phase IIa Study of the Product QGC001 Compared with Placebo in Patients with Essential Hypertension (2QG1) NCT02322450 [48,49]	A pilot multicenter double-blind randomized placebo-controlled crossover pharmacodynamic study. 34 participants Phase IIa	Firibastat was studied for 4 weeks in patients with primary mild HT. Daytime ambulatory SBP and office SBP decreased by 2.7 and 4.7 mmHg, respectively, compared to placebo. No major side effects were observed. Firibastat had no effect on heart rate, plasma renin, aldosterone, apelin and copeptin concentrations during the 4 weeks. AEs: 14 patients with 16 reversible adverse events of mild intensity. One case of withdrawal due to rash, vestibular disorder and arthralgia each.	Treatment with firibastat in hypertensive subjects over 4 weeks reduced their SBP relative to placebo. The systemic RAS was not affected. These results allowed larger, longer, and more powerful clinical trials to fully explore the efficacy of firibastat.
Novel Evaluation with QGC001 in Hy- pertensive Overweight Patients of Multiple Ethnic Origins (NEW-HOPE) NCT03198793 [44,50]	Open-Label Dose-TitratingSafety and Efficacy Study 256 participants Phase IIb	For 8 weeks, the effect of twice daily administration of oral firibastat among hypertensive overweight/obese people of different races has been investigated. Firibastat reduced office SBP by 9.5 mmHg and office DPB by 4.2 mmHg. Significant de- creases in BP were found in all subgroups. Among obese people, SBP decreased by 10.2 mmHg, 10.5 mmHg among blacks and 8.9 mmHg among non-blacks. AEs: 14.1% related treatment-emergent AEs (headache, skin reactions most common), one serious related AE (erythema multiforme) resulted in discontinuation	Firibastat is effective in lowering the BP, also in the high-risk groups. Firibastat is a possible alternative when it comes to difficult-to-treat patients or resistant hypertension where monotherapy with standard treatment options is no longer effective.
Firibastat in Treatment-resistant Hypertension (FRESH) NCT04277884 [51]	Double-blind, Placebo-controlled, Efficacy and Safety Study 502 participants Phase 3	This study is still recruiting and has not been completed yet. For that reason, the results are missing from this study. The objective of this study is to investigate the effect of firibastat in people ≥18 years with uncontrolled primary hypertension. Administrations of firibastat 500 mg orally twice daily over 12 weeks are compared with placebo.	No conclusions can be drawn since the study is not completed.
Randomized Study of Extended Treatment with Firibastat in Treatment-Resistant Hypertension. (REFRESH) NCT04857840 [52]	Double-blind, Placebo-controlled and Open-label Efficacy and Long-term Safety Study 750 participants Phase 3	This study is still recruiting and has not been completed yet. The objective is to investigate the efficacy and safety of 1000 mg (2 × 500 mg p.o.) firibastat in addition to their chronic antihypertensive therapies for up to 48 weeks in patients with difficult-to-treat/treatment-resistant HTN	No results have been reported yet

**Table 3:** Search terms and number of hits (overview).

Keyword	Number of Hits
Hypertension	577,486
Hypertension and treatment	322,085
Arterial hypertension and treatment	310,742
APA inhibitor	643
Brain RAS and hypertension	421
EC33	32
RB150	30
APA inhibitor and Ang-III	25
Firibastat	23
Quantum Genomics and firibastat	12
QGC001	3

## Data Availability

Data sharing not applicable.

## Abbreviations

ABPM	Ambulatory blood pressure measurement
ACE	Angiotensin converting enzyme
AE(s)	Adverse effect(s)
Ang-II	Angiotensin-II
Ang-III	Angiotensin III
Ang-IV	Angiotensin IV
APA	Aminopeptidase A
APN	Aminopeptidase IV
ARB(s)	Angiotensin-II-receptor blocker(s)
AT1	Angiotensin type 1 receptor
AT2	Angiotensin type 2 receptor
AVP	Arginine-vasopressin
BP	Blood pressure
CVD	Cardiovascular disease
DBP	Diastolic blood pressure
DOCA	Deoxycorticosterone acetate
EH	Essential hypertension
HBPM	Home blood pressure measurement
HT	Hypertension
RAAS	Renin–angiotensin–aldosterone system
RAS	Renin–angiotensin system
SBP	Systolic blood pressure
SH	Secondary hypertension
SHR	spontaneously hypertensive rat
SNS	Sympathetic nervous system
WHO	World Health Organization
